# Understanding community-based participatory research through a social movement framework: a case study of the Kahnawake Schools Diabetes Prevention Project

**DOI:** 10.1186/s12889-018-5412-y

**Published:** 2018-04-12

**Authors:** Marie-Claude Tremblay, Debbie H. Martin, Alex M. McComber, Amelia McGregor, Ann C. Macaulay

**Affiliations:** 1 Department of Family Medicine and Emergency Medicine, Office of Education and Continuing Professional Development, Université Laval, 1050, de la Médecine, Pavillon Ferdinand-Vandry, 2881-F, Québec, QC G1V 0A6 Canada; 2 School of Health and Human Performance, Dalhousie University, Halifax, NS Canada; 3 Kahnawake Schools Diabetes Prevention Project, Kahnawake, QC Canada; 4 Department of Family Medicine, McGill University, Montreal, QC Canada

**Keywords:** Community-based participatory research, Social movements, Collective action, Process evaluation, Program evaluation, Indigenous health, Health promotion, Diabetes prevention

## Abstract

**Background:**

A longstanding challenge of community-based participatory research (CBPR) has been to anchor evaluation and practice in a relevant theoretical framework of community change, which articulates specific and concrete evaluative benchmarks. Social movement theories provide a broad range of theoretical tools to understand and facilitate social change processes, such as those involved in CBPR. Social movement theories have the potential to provide a coherent representation of how mobilization and collective action is gradually developed and leads to systemic change in the context of CBPR. The current study builds on a social movement perspective to assess the processes and intermediate outcomes of a longstanding health promotion CBPR project with an Indigenous community, the Kahnawake Schools Diabetes Prevention Project (KDSPP).

**Methods:**

This research uses a case study design layered on a movement-building evaluation framework, which allows progress to be tracked over time. Data collection strategies included document (scientific and organizational) review (*n* = 51) and talking circles with four important community stakeholder groups (*n* = 24).

**Results:**

Findings provide an innovative and chronological perspective of the evolution of KSDPP as seen through a social movement lens, and identify intermediate outcomes associated with different dimensions of movement building achieved by the project over time (mobilization, leadership, vision and frames, alliance and partnerships, as well as advocacy and action strategies). It also points to areas of improvement for KSDPP in building its potential for action.

**Conclusion:**

While this study’s results are directly relevant and applicable to the local context of KSDPP, they also highlight useful lessons and conclusions for the planning and evaluation of other long-standing and sustainable CBPR initiatives. The conceptual framework provides meaningful benchmarks to track evidence of progress in the context of CBPR. Findings from the study offer new ways of thinking about the evaluation of CBPR projects and their progress by drawing on frameworks that guide other forms of collective action.

**Electronic supplementary material:**

The online version of this article (10.1186/s12889-018-5412-y) contains supplementary material, which is available to authorized users.

## Background

Community-based participatory research (CBPR) is an approach to research that involves collective, reflective and systematic inquiry in which researchers and community stakeholders engage as equal partners in all steps of the research process with the goals of educating, improving practice or bringing about social change [1–3]. At its core, CBPR questions the power relationships that are inherently embedded in Western knowledge production, advocates for power to be shared between the researcher and the researched, acknowledges the legitimacy of experiential knowledge, and focuses on research aimed at improving situations and practices [3]. This approach to research is recognized as particularly useful when working with populations that experience marginalization – as is the case for some Indigenous communities—because it supports the establishment of respectful relationships with these groups, and the sharing of control over individual and group health and social conditions [3, 4].

A longstanding challenge of CBPR has been to anchor evaluation and practice in a relevant and comprehensive theoretical framework of community change [4–8]. Given the complex causal web linking CBPR projects to specific health outcomes, traditional measurement strategies may neither be sensitive enough nor adequate to assess change and document successes or failure at the community level [6, 9, 10]. In addition, our understanding of the processes that link community-based collaborative action to changes in systemic determinants of health outcomes is still limited [6, 8]. To date, most evaluative frameworks of CBPR have focused on the internal characteristics of coalitions and partnerships [7, 11], provided general guidance on implementation steps [8, 12] or used logic models to map out desired outcome categories [13]. There is a need to articulate specific, concrete and sequential evaluation benchmarks for CBPR in a detailed and theoretically consistent framework [6].

Social movements, generally viewed as large group actions that promote social change [14, 15], share a set of common features with CBPR, such as aiming to reverse unequal relations of power by creating broad social, policy and systemic changes [4, 16, 17]. The field of social movement research has produced a vast array of theoretical approaches, providing substantial theoretical tools to understand and facilitate collective action and social change [14, 15, 18–21]. While many fields of research and action aimed at social betterment have been inspired by social movements [10, 22, 23], to our knowledge social movement theories have never been explicitly used to inform and better understand CBPR processes. We believe these theories can provide a coherent representation of how mobilization and collective action is gradually developed and leads to systemic change in the context of CBPR.

As a first step in assessing the relevance of social movement theories to understanding CBPR, we conducted a framework synthesis of illustrative CBPR projects (8) using a multidimensional social movement theory-based framework [24]. This synthesis, presented elsewhere [24], resulted in the development of a multidimensional framework through which to conceive and map community change processes in the context of CBPR. In addition, our synthesis demonstrated the relevance of using modern social movement theories, such as resource mobilization theory [15, 20, 25, 26], political process theory [14, 20, 21, 27] and framing theory [14, 28–30], to understand and examine CBPR processes. More specifically, it demonstrated that CBPR projects, like social movements, can be envisioned as collective processes evolving dynamically and iteratively through a four-stage lifecycle: (1) emergence, (2) coalescence, (3) momentum, (4) maintenance, consolidation, integration or decline. Key elements of this four-stage process include capitalizing on resources, opportunities, and building partnership and collaboration among different organizations and entities. Just like a social movement, CBPR also makes strategic use of collective framing processes to define a representation of a social problem (cause), mobilize around the cause as well as to define a collective action strategy leading to system changes addressing the problem [24]. Here, we draw on the conclusions of our previous work to design and evaluate a specific CBPR project.

### Purpose of the study

The goal of the current study is to assess the community-level processes and intermediate outcomes of a longstanding CBPR initiative developed with an Indigenous community, the Kahnawake Schools Diabetes Prevention Project (KSDPP), using a social movement theory perspective. More specifically, this research builds on a movement-building evaluation framework to assess the general process underlying KSDPP as well as intermediate outcomes related to core movement-building concepts. In keeping with the purpose of most evaluative research, this study aims to provide results that are directly relevant and applicable to KSDPP, but also to highlight useful lessons for CBPR planning and evaluation more broadly.

### Conceptual framework

There are a range of evaluative frameworks and benchmarks used to assess social movement building, advocacy efforts and policy-change action [31–33]. Amongst them, Master and Osborn’s [31] comprehensive framework, which builds on a literature review of outcomes associated with social change, is particularly relevant for this study. Whereas many existing evaluative frameworks only provide end-of-project benchmarks, Master and Osborn’s framework provides a general perspective of how social movements can be conceived and allows for an meaningful exploration of movements’ development over time. This framework appeared particularly relevant to synthesize the most important concepts of social change.

Master and Osborn’s framework incorporates intermediate outcomes of five core components of movement building: base building and mobilization, leadership, vision, alliances, and advocacy infrastructure (Table 1). Each of these five components develop across four stages of movement building, facilitating a comprehensive and dynamic portrayal and assessment of a movement’s evolution over time. This comprehensive array of intermediate outcomes at different stages of a collective action process (distinct from impact outcomes related to a movement’s activities) are useful in the assessment of the development of a CBPR project over time.

**Table 1:** Master and Osborn’s [31] movement building evaluation framework

Stages/Core components	Base building and mobilization	Leadership	Vision and frames	Alliances, partnerships, networks	Advocacy agenda and action strategy
Stage 1 - Emergence	Participation of both paid and volunteer leaders is developed in base-building organizations; Reflection time and assessment are built into the movement activities	Movement leaders and the roles they play emerge and are recognized within the movement; Leaders are supported to develop their skills, roles and visibility	A process for creating a shared analysis of the problem is developed; Movement organizations develop strategic plans with explicit movement goals	Alliance anchors increase organizations capacity; Capacity for collaboration is developed	Needed skills and organizational capacities are identified and developed
Stage 2 - Coalescence	New leaders are recruited; New members and constituencies are recruited and the base is expanded	Collaborative leadership philosophy is widely adopted by movement leaders; Leaders of the movement are respected for their different roles and responsibilities within the movement	Movement leaders develop shared values, motivations, and interests; Movement values and priorities begin to gain salience outside of the movement	Number, breadth, and capacity of alliances are strengthened; Joint strategic planning and identification of priorities among anchor organizations occurs; Trust is built among alliance members	Identification of collective action goals; Collaborative fundraising and sharing of resources increases
Stage 3 - Movement’s moment	Power and leadership of the movement are recognized by the community base; Movement experiences rapid recruitment and growth	Movement leaders are recognized by public institutions and political institutions	Public support of the meta-narrative increases; Political will for movement goals significantly increases	Movement organizations share resources; Movement builds relationships with other movements	Major initiatives advance and are implemented; Collective action reaches a peak
Stage 4 - Maintenance, integration, consolidation	N/A	New generation of leadership emerges	Norms change and the vision becomes widely shared among public and political leaders	N/A	Movement’s priorities and advocacy agenda are widely accepted and continue to drive agendas of movement organizations

### The Kahnawake Schools Diabetes Prevention Project

Kahnawake is a north-eastern Kanien’kehá:ka (Mohawk) community of 7859 residents (2017) that is situated on the south shore of the St. Lawrence River, 10 miles from downtown Montreal (Quebec, Canada). The Kanien’kehá:ka are part of the Haudenosaunee, or “People of the Longhouse”, historically known as the Five Nations, or Six Nations Iroquois Confederacy. Traditional and cultural Haudenosaunee values emphasize collective thinking, shared responsibility, listening, taking into account the impact of current decisions on future generations, consensus decision-making, as well as a wholistic view of health, all of which provide a fertile ground for developing a CBPR project [34]. As a community, Kahnawake has demonstrated independence and autonomy in many domains, resulting in decentralization in the provision of a number of community services such as education, health, youth recreation programs for youth, and social services.

Despite this history of strength and independence, Kahnawake has been transformed by Western colonization, which has created social conditions that promote poorer food and lifestyle choices [35]. In 1985, two family physicians working in Kahnawake perceived high rates of Type 2 diabetes, and conducted a study to assess the prevalence of this condition in the community. Findings from the study showed that 12% of adults aged 45–64 had Type 2 diabetes, which was twice the rate of the general population [36]. Study findings also showed a high prevalence of diabetes related complications [37, 38]. Based on these results, the physicians made a series of community presentations that raised awareness about diabetes, and shifted perceptions relating to the preventability of this disease [39]. Acting on this new awareness, community leaders mobilized and sought the expertise of academic researchers to develop a diabetes prevention program which became the Kahnawake Schools Diabetes Prevention Project (KSDPP), a CBPR project with a high degree of community involvement and ownership [40–42].

KSDPP aims to change the physical environment and social norms of the schools and community by promoting healthy eating and regular physical activity not only among children, but also parents, teachers, and all community members [43, 44]. The project initially developed around a school-based component bolstered by community outreach interventions. The school-based component originally consisted of a health education curriculum delivered by teachers in Kahnawake elementary schools and a nutrition policy promoting healthy food choices at school. This policy was later expanded to include the promotion of physical activity and a whole range of healthy lifestyle activities. Community interventions include a variety of activities, many conducted in partnership with community organisations. The central goals of the community interventions are to create environments that support behavior change through activities tailored for parents, grand-parents and other community members [34, 43]. While the program of activities is anchored in evidence-based theories of behavior and community change, the core of KSDPP’s actions are based on Kanien’kehá:ka values and traditions, and a wholistic view of health which incorporates the physical, emotional, mental and spiritual dimensions of life, true to a Haudenosaunee perspective of well-being [34, 45]. For instance, the intervention’s primary target is elementary school children, which is consistent with the Kanien’kehá:ka value of taking responsibility to protect and promote the health of present and future generations (Seven Generations) [43]. The general approach of building supportive environments for health is in line with the Kanien’kehá:ka wholistic approach to education which takes into account the broader environment in which children develop [46]. In addition, KSDPP’s style of governance is deeply rooted in Kanien’kehá:ka values, which involve consensus in decision-making and a collective vision for the community [43].

Since the project’s inception, many studies have attempted to evaluate the impact of KSDPP on the health status and lifestyles of residents in the community. These studies have shown mixed results in the areas of physical activity, nutrition, weight and rates of diabetes [47–51]. The present study applies social movement concepts to expand and enrich this examination by identifying intermediate outcomes of KSDPP in the area of community mobilization and change, dimensions that are viewed as highly relevant and meaningful by KSDPP stakeholders. The goal of this research evaluation project was to develop a new understanding of KSDPP’s evolution, identify potential areas of improvement, and action paths for further mobilization of community workers and members around the issue of diabetes prevention. Results of the study were meant to inform the work of KSDPP and the greater Kahnawake community.

## Methods

### Research approach and design

We used a case study design, which is a systemic approach to qualitative research that allows the researcher to examine in depth the holistic nature of contemporary phenomena in natural contexts, with a multitude of data sources [52, 53]. The case observed is the *Kahnawake Schools Diabetes Prevention Project* (KSDPP), bounded in time from its first ideation (around 1987) to present.

In accordance with KSDPP principles, this study builds on a community-based participatory approach, involving partnership building, regular exchange among partners, and experience sharing between the researchers, KSDPP intervention staff and the Community Advisory Board (CAB) [54]. This study uses an interpretivist perspective, which holds that reality is constructed through the meanings developed by social actors, including the investigators. Thus, findings emerged through dialogue and negotiation of interpretations between the researchers and stakeholders involved in this study.

In 2012, the first author approached KSDPP to explore their interested in the innovative idea of evaluating the community level processes and outcomes of KSDPP using social movement theories. As a result, the first author was invited to join the KSDPP research team as a postdoctoral investigator, attend monthly meetings of the CAB and the research team, and to engage in KSDPP activities and with the community of Kahnawake. As a settler, the first author did not have any previous research experience in partnership with an Indigenous community, and therefore sought to immerse herself in the culture and realities of the community. During her work, she was supervised by and benefited from the valuable advice, insight and knowledge of community leaders (AMG and AMC). The research proposal was designed and developed in full partnership with the KSDPP team to ensure cultural relevancy, and benefits for both KSDPP and the broader community. Stakeholders were involved in developing the research questions and methodology, as well as in data collection, the interpretation of findings and dissemination of results.

### Data collection

Two data collection strategies were used in this case study (1) document review and (2) talking circles with four important stakeholder groups (data sources are described in Table 2).

**Table 2:** Data sources

Data collection strategies	Data sources	Descriptions	*N*
1. Document review	Documents types		*n*
	1. KSDPP annual summaries of activities and work plans	Description of school- and community-based program of activities, from year 1994 to 2016	12
	2. Scientific publications	Publications in academic journals, thesis and book chapter directly related to KSDPP (including descriptions of design and general approach of the project, implementation evaluation, outcomes assessment) or related to the antecedent stage of KSDPP (for instance, publications documenting baseline rates of diabetes in Kahnawake), from year 1988 to 2016.	39
Total documents			51
2. Talking circles	Stakeholders groups		*n*
	1. Intervention staff and Community Advisory Board (CAB) members	Past and current KSDPP intervention staff who develop(ed) and implement(ed) KSDPP health promotion interventions in the schools and the community. Community Advisory Board (CAB) members are past and current members of the committee supervising the administrative and financial operations of KSDPP, reviewing all intervention, research and training activities and ensuring research accountability to the community.	7
	2. Research team members	Past and current community researchers from Kahnawake and researchers from various universities (including Université de Montréal and McGill University) that have contributed to a research project with KSDPP.	7
	3. Community workers	Professionals working in different public sectors of the community (education, healthcare and social services) and providing direct or indirect services to or for the benefit of community members.	5
	4. Community members	Residents of Kahnawake who are not involved in the previous groups and that can be conceived more as potential beneficiaries of the program (children’s relatives including parents and grand-parents).	5
Total participants			24

Included in the review were documents that provided a comprehensive portrait of KSDPP’s evolution since 1994 in terms of key aspects of collective action such as leadership, community mobilization, KSDPP’s discourse and meta-narrative, alliance and partnerships, as well as program of activities. Documents reviewed were past and current KSDPP summaries of activity or work plans covering the years 1994 to 2016 (*n* = 12), as well as published scientific papers stemming from the project (*n* = 39). Organizational documents dating from before 2006 were only available in paper format and were digitized. Scientific publications that included KSDPP as one of a number of cases and published abstracts were discarded (*n* = 6), since these publications only provided shallow descriptions of KSDPP and redundant information. A list of all included publications is presented in Additional file 1. Scientific and organizational documents were collected in January 2016 through direct solicitation, or downloaded from KSDPP and the research team websites (ksdpp.org; pram.mcgill.ca) as well as a bibliographical database.

Talking circles are widely used to collect data in many Indigenous contexts, offering a means to collect data that encourages story-telling and collective listening – both important elements for sharing and gathering information within Indigenous contexts. Importantly, talking circles have been accepted by the Kahnawake community as a relevant data collection strategy. In a talking circle, participants sit in a circle and discuss specified topics until consensus is reached. An object (an eagle feather, a talking stick or a stone), is passed from one participant to another and the holder of the object has an opportunity to speak [55]. Talking circles were deemed useful in gathering stakeholder perceptions about the evolution of KSDPP, its collective action process and strategies, leadership, vision and partnerships. They also served to document the last stage of the project given the dearth of scientific publications after 2009. A talking circle guide, informed by the conceptual framework, was developed in partnership with the KSDPP team. This guide had questions about: (1) the importance of diabetes for the community; (2) the evolution of mobilization around diabetes in the community over the last 20 years; (3) community leaders (people or organizations) involved in diabetes prevention (4) perception of KSDPP and its impact over the last 20 years; (5) KSDPP’s vision (goal) (6) evolution of KSDPP’s action (7) community partners and collaborators of KSDPP; (8) strengths of KSDPP and actual challenges for diabetes prevention.

Participants involved in the study talking circles (*n* = 24) were also KSDPP stakeholders, i.e. individuals or groups with a vested interest in the focus of the evaluation or research [56]. They included: (1) KSDPP intervention staff and Community Advisory Board (CAB) members; (2) research team members; (3) community workers; (4) community members (see Table 2 for a full description). Recruitment of talking circle participants proceeded on a voluntary basis. Participants in the first two circles were recruited through a formal email invitation sent to current and past KSDPP staff members, CAB members and researchers, one month prior to the beginning of the study (the KSDPP team assisted in the creation of the lists). Participants in the remaining circles were recruited using general invitations mailed directly to a list of partner organizations created by the KSDPP team, announcements in the local newspaper, and direct solicitation of community members at community events, such as community walks.

In total, 5 talking circles were held between October and December 2015, each including 2 to 7 participants. In general, there was one talking circle for each stakeholder group, except the community worker group (group 3), which required 2 talking circles to fit the availability of participants. Talking circles were held in community facilities (community rooms and schools) over lunchtime to accommodate participants. Participants were provided with a light meal, which is a culturally appropriate manner in which to thank them for their participation. The average length of the talking circles, including the time spent explaining the study, was 2 h (range 1 h to 2 h 20 min). Talking circles provided a respectful and ordered structure through which to collect in-depth data, triangulate information, and build a common representation of events and times. Consensus was achieved when everyone felt that they could agree with the suggested statement. Following Kanien’kehá:ka decision making style, all participants came to ‘one mind’ as close as possible, all agreed to have a voice in the discussion.

### Ethics approval and consent to participate

As with all KSDPP research projects, this project was conducted in accordance with the KSDPP Code of Research Ethics [57], which serves as a binding research agreement between the researchers and the community. Ethical approval was obtained first from the CAB and then from the McGill University ethics institutional review board. Participants in the talking circle provided individual written informed consent.

### Data analysis

The analytic technique used in this study is framework analysis, a method for analysing primary data in applied social research that draws upon the work of Bryman and Burgess [58] and Miles and Huberman [59]. Framework analysis is useful for synthesizing knowledge from diverse sources [60]. This analysis technique typically involves five phases [61]: (1) familiarisation with the data; (2) identification of a relevant thematic framework; (3) application of the thematic framework by indexing all the data to specific themes; (4) organization of the data according to themes in a chart containing distilled summaries of views and experiences; (5) interpretation of findings, which involves mapping the range and nature of phenomena, creating typologies and finding association between themes.

Hard copies of publications (mostly organizational documents dated 2005 or earlier) were scanned and converted to PDF. All talking circles were audio recorded and transcribed verbatim. To perform the analysis, a database including all sources of data (full-text scientific papers, organisational documents, and transcripts from the talking circles) was constructed using QSR NVivo 11 [62]. Using the framework analysis method, the first author immersed herself in the data, identifying key ideas (mobilization, leadership, goal and vision, collaboration and partnership, activities and strategies), and then searched the literature for a relevant thematic framework. Our work in this phase was informed by the results of a framework synthesis we conducted previously that demonstrated the relevance of modern social movement theories in the study of CBPR projects [24]. For the current study, we chose to use Master and Osborn’s movement-building framework, which provides a means to examine the development of various components of social movements over time. Based on Master and Osborn’s framework, the first author developed a coding grid and performed sentence by sentence coding to assign text to specific themes (components and stages). At this stage, we also added an inductive component building on thematic analysis to identify potential new themes from the data [59]. All coded material was organized in a chart presenting summaries of views and experiences for each theme, and facilitating a comprehensive interpretation of KSDPP process and intermediate outcomes in terms of movement building.

The first author conducted the majority of the analysis, but all provisional interpretations were discussed with the KSDPP research team, staff and CAB members. Two formal data interpretation sessions were held to discuss interpretations, add context to information collected, and facilitate a better understanding of project documentation. For instance, during these sessions participants built consensus on the start and end dates of each stage, as well as markers of change for each period (referred to as “benchmarks” in the framework). The resulting interpretation was therefore consensual and co-created by the different team members. Construct and internal validity of the study were ensured by triangulation of data sources and methods, member checking, and the in-depth involvement of the researcher in the field. Finally, reliability of the study was improved by the development and use of a case study protocol and the development of database and a chain of evidence [52].

## Results

Results show an innovative and chronological perspective of KSDPP’s evolution as seen through a social movement lens, as well as intermediate outcomes associated with different dimensions of movement building achieved by this project over time. The inductive component of the analysis suggests new benchmarks pertaining to some movement-building components (bolded in the table). The dates proposed for each stage are approximate and should be understood as temporal benchmarks, as phases often overlap.

The next section outlines the different stages of KSDPP in narrative style, describing the important benchmarks reached, which are summarized in Table 3.

**Table 3:** Results: KSDPP’s evolution in terms of movement-building benchmarks

Stages	/Core components	Base building and mobilization	Leadership	Vision and frames	Alliances, partnerships, networks	Advocacy agenda and action strategy
Stage 1 – KSDPP’s emergence (Early 1987 – mid-1997)	*Benchmarks*	*- Participation of both paid and volunteer leaders is beginning in base-building organizations;* *- Reflection time and assessment are built into the movement activities*	*- Movement leaders and the roles they play emerge and are recognized within the movement;* *- Leaders are supported to develop their skills, roles and visibility*	*- A process for creating a shared analysis of the problem is developed;* *- Movement organizations develop strategic plans with explicit movement goals;*	*- Alliance anchors increase organizations capacity;* *- Capacity for collaboration is developed*	*- Needed skills and organizational capacities are identified and developed*
	Evidence of achievement	- Kahnawake community leaders and elders start to mobilize and seek the collaboration of academic partners. - The Community Advisory Board (CAB) is formed with more than 40 volunteering people, representing a wide spectrum of local organisations, services, and the community at large. - All the partners take the time to collectively develop the project’s vision and the terms and conditions of the partnership.	- Community leaders, including elders and family physicians that raised the alarm about diabetes, invite academic researchers to join the partnership for their expertise in community research. - The intervention team is staffed by two full-time community members, chosen for their leadership in the community. These staff members can acquire new skills and enhance their competencies through formal training.	- In the early beginnings, a community awareness process allows to shift the perception of diabetes from a matter of fact to a community issue that can be acted on. - After acquiring funding, the partnership defines a shared vision that proposed an ideal for Kahnawake and lays the ground for strategic goals. - The terms and conditions of the participatory research process are collaboratively developed through a Code of Ethics.	- KSDPP developed from the alliance of community-based professionals coming from the Kahnawake Education Centre and the Kateri Memorial Hospital Centre, as well as researchers from McGill University and Université de Montréal. - The newly formed CAB included volunteers from multiple sectors of the community, that increased capacity for collaboration with local health, education, recreation, and community service organisations.	- Formal training in various areas for community and staff members develop new skills and increased capacities. - KSDPP provided opportunities for community collaborators to acquire new skills (e.g. new health curriculum delivered by the teachers).
Stage 2 – KSDPP’s coalescence (mid-1997 - 2000)	*Benchmarks*	*- New leaders are recruited;* *- New members and constituencies are recruited and the base expand*	*- Collaborative leadership philosophy is widely adopted by movement leaders;* *- Leaders of the movement are respected for their different roles and responsibilities within the movement*	*- Movement leaders develop shared values, motivations, and interests;* *- Movement values and priorities begin to gain salience outside of the movement*	*- Number, breadth, and capacity of alliances are strengthened;* *- Joint strategic planning and identification of priorities among anchor organizations occurs;* *- Trust is built among alliance members*	*- Identification of collective action goals;* *- Collaborative fundraising and sharing of resources increase*
	Evidence of achievement	- Important community leaders (the Mohawk Council of Kahnawake, Kahnawake Shakotiia’takehnhas Community Services, and the Kahnawake Education Center) commit to KSDPP and provide funds to enable KSDDP’s action. - Teachers began to be more comfortable with the new curriculum and are committed to the cause of KSDPP.	- KSDPP implements a participatory /collaborative and non-hierarchical style of governance. - Respectful relationships of collaboration are established between partners.	- KSDPP translates its vision into a full and workable action strategy that build and integrate traditional and cultural values. - The fact that community partners provide funds to KSDPP is a good indicator of KSDPP’s values and priorities being accepted and prioritized by community actors.	- At that time, half of activities are conducted by KSDPP independently whereas half result from collaborative partnerships between KSDPP and partners. - Community members and organisations bring in their knowledge of the community, and contribute ideas on how best to carry out the activities in which they are involved. - Trust and respect characterize the relationships with the education system.	- The intervention team establishes core of intervention activities and develops a good experience in implementing these within the community. - Collaboration allows partners to optimize community resources, share responsibilities and support each other’s efforts.
Stage 3 – KSDPP’s moment (2001–2006)	*Benchmarks*	*- Power and leadership of the movement are recognized by the community base;* *- Movement experiences rapid recruitment and growth*	*- Movement leaders are recognized by public institutions and political institutions*	*- Public support of the meta-narrative increases;* *- Political will for movement goals significantly increases;* *- Proposed benchmark: Increasing dissemination of the program vision and goals*	*- Movement organizations share resources;* *- Movement builds relationships with other movements;* *- Proposed benchmark: Movement enlarges its scope*	*- Major initiatives advance and are implemented;* *- Proposed benchmark: Collective action reaches a peak*
	Evidence of achievement	- During this stage, KSDPP benefits from deep community roots and recognition. - Kahnawake is recognized as a diabetes prevention leader among First Nations communities across Canada and internationally.	- KSDPP secures funding to develop the KSDPP Center for Research and Training in Diabetes Prevention, this acknowledging KSDPP experience, expertise and leadership in this field. - KSDPP staff and research team members are elected at important positions in national diabetes and international research networks.	- KSDPP becomes more active and extensively spread its vision locally and nationally through participation in national forums addressing diabetes and health issues for Indigenous people. - The newly created Onkwatakaritatshera health research council acknowledges KSDPP’s CAB as a valid and autonomous ethics authority for diabetes research prevention and adds KSDPP’s code of research ethics to their toolkit.	- KSDPP’s program of activities, already collaborative in nature, continues to build on partners’ strengths, allowing to increase both the reach and intensity of the program. - KSDPP’s program expands to include preschool children and engages adolescents in youth empowerment projects. - Partnerships with local organizations broaden to include local businesses.	- KSDPP’s collective action strategy reaches a peak, building on a core program of activities that has achieved maturity and the addition of other activity components. - More than 100 different interventions target individuals of all ages, families, organizations and political groups.
Stage 4 – KSDPP’s maintenance and integration (2007 - now)	*Benchmarks*	*- Proposed benchmark: Mobilization slows down and the movement appears less visible*	*- New generation of leadership emerges and builds capacities*	*- Norms change and the vision becomes widely shared among public and political leaders*	*-* *Proposed benchmark* *:* *Capacity-building of the partners allows for transfer of responsibilities*	*- Movement’s priorities and advocacy agenda are more widely accepted and continue to drive agendas of movement organizations and partners*
	Evidence of achievement	- Decrease in resources, coupled with a lack of novelty, rendered KSDPP less visible. - Community mobilization slowly decreases. - Some administrative environments in the community become less sensitive to KSDPP’s action.	- A new generation of leaders in different components of the partnership, including KSDPP staff and research team, is slowly emerging.	- The vision promoted by KSDPP (a healthy community free of diabetes) and the norm underlying this vision (diabetes is a preventable disease) are adopted by many community members.	- Community partners are now taking over some of the responsibilities initially held by KSDPP (e.g. school physical activity policy, active school transportation project).	- KSDPP’s agenda is integrated into those of some partnering organisations, such as schools.

### The emergence of KSDPP: from early 1987 to mid-1997

The first stage of KSDPP, which we call emergence, began in 1987 when community leaders first evoked the idea of developing an intervention to prevent type 2 diabetes in Kahnawake [39].

The first stage emerged following a shift in the perception of diabetes following a lengthy community awareness-building process implemented from the mid- to late-1980s [39, 43]. During this process, baseline research results were shared with the community shifting the perception of diabetes from being a personal issue to a community issue. The idea that diabetes could be prevented was slowly articulated in the late 1980s and early 1990s [39].

Volunteer community leaders, including elders and family physicians who raised the alarm about diabetes, invited academic researchers with expertise in community research to join the effort of elaborating a project proposal and developing a partnership [43]. After a few unsuccessful attempts, the team secured national research and intervention funding in 1994, and formally initiated the project [41]. One of the early exercises of the team consisted in elaborating operating guidelines and conditions for the participatory research process underlying KSDPP through a Code of Ethics [43, 57]. “The process of creating a KSDPP partnership involving community researchers, academic researchers, and the community has been facilitated and strengthened by the joint development of a Code of Research Ethics during the first year of the project” [41].

The underlying philosophy of KSDPP (a participatory research process) was easily implemented because it converged with a Kanien’kehá:ka tradition of consensus decision-making [43]. At the same time, the partners also defined an inspirational and shared vision for Kahnawake that portrayed a community free of diabetes, living healthily and in wholistic balance. This vision, which laid the ground for the elaboration of strategic goals, was framed according to important cultural values of the Kanien’kehá:ka, such as a collective concern for the welfare of future generations (Seven Generations) and a wholistic philosophy of health [34]. As mentioned by one talking circle participant, in the first stage of KSDPP, collective reflection around the project, its goals and processes was highly important and helped set the stage for future steps:

“It took a year, a year and a half to prepare things once we had the grant. I remember saying things like ‘We need to do things, it takes time that we are out there. If we want to have an effect, we need to do things’. So we did such things as developing a code [of research ethics], a vision, developing all those kinds of things that take a lot of time, take a lot of discussion of participatory nature (…). I think that the way we did things put a very solid foundation; that what is sustained there, this kind of vision, this kind of relationship, the code of research ethics, and those kinds of things are traceable through those times.” (group 2)

KSDPP developed from a partnership that was initially formed through an alliance of professionals from the Kahnawake Education Centre, the Kateri Memorial Hospital Centre and Kahnawake Shakotiia’takehnhas Community Services (social family services), as well as researchers from McGill University and Université de Montréal. A talking circle participant (group 1) discussed the importance KSDPP’s roots in community: “I think that the grassroots connection that KSDPP has from the beginning is a very important strength. It’s the people from the community that… we, people in the community who are associated with KSDPP”. Over the first three years, the partnership recruited around 40 volunteers from multiple local organizations who formed the KSDPP Community Advisory Board (CAB) [43]. This CAB was (and is still) responsible for supervising all aspects of the project, from the design of the intervention through implementation and assessment. Through this new structure, “partnerships among local health, education, recreation, and community service organisations were formed, enhancing community participation” [41] as well as collaborative leadership.

In the first years of program implementation (1994–1997), the intervention team was staffed by two full-time community members, selected for their leadership and their role as agents for change [43]. As evoked by a talking circle participant (group 3), the choice of these persons was strategic, because they “came from the education system, so not only they were from the community but they were teachers so everyone knows them in that circle”. These staff members participated in formal training activities in order to acquire new skills in health promotion or enhance their competencies [41]. The program also provided many opportunities for collaborators to acquire new competencies. For instance, KSDPP supported the implementation of a new health curriculum in the elementary schools. While the curriculum was created by nurses and a nutritionist it was developed to be delivered by teachers (as opposed to health care professionals) who assumed full responsibility for the program in 1997 [46].

### Coalescence of KSDPP: from mid-1997 to 2000

Beginning in August 1997, KSDPP experienced a series of events prompting the partnership to reinforce, take shape and deepen its ties in the community.

As the initial 3-year intervention and research grant was coming to an end in mid-1997, KSDPP began to seek new sources of support [41]. In June 1997, community partners (the Mohawk Council of Kahnawake, Kahnawake Shakotiia’takehnhas Community Services, and the Kahnawake Education Center) provided funds to enable the project to continue for one year (1997–1998) (funding was for the intervention component of KSDPP) [63]. These new funding partners, who were essentially new constituencies, were fully committed to the project. For talking circle participants (group 1), the fact that community partners provided funds for KSDPP to continue is an indicator of the value given to KSDPP by community stakeholders, who “were highly mobilized by the cause and pooled resources”. Following the year of community funding, continuing funds were secured from external private foundations (1999–2001).

Already at this stage, the participatory decision-making process and collaborative governance of the project were well established. In fact, study findings for that period point to a participatory democracy or non-hierarchical decision-making process as the primary mode of KSDPP governance [42, 64]. For instance, it was reported that “The influence of multiple partners in determining the overall direction of KSDPP demonstrates the responsiveness and accountability of the egalitarian leadership style promoted by project staff” (p. 184) [64]. In addition, in one of the talking circles (group 3), a participant from a community organization and former CAB member described the way KSDPP invited partners to join the CAB, emphasizing the leadership style that KSDPP put in place:

“(KSDPP) went up there, spoke and invited people to come and sit on the Community [Advisory] Board… [this] was a place where your ideas were acceptable. Like you had to be the ones to write the terms of reference, you had to be the one for this mission, (...) it was always like a corporate thing.”

KSDPP’s coalescence was characterized by the translation of KSDPP’s vision into a full and workable action strategy that builds on, and integrates traditional and cultural values: “Activity implementation was embedded within an overall program intervention cycle directed towards promoting living in balance, in turn, a reflection of local cultural values” [34]. Living in balance, which “reflects being well in mind, body, emotion, and spirit” [34] is congruent with the Haudenosaunee wholistic approach of health [34, 46]. By 1997, the team had established the core intervention activities and had experience implementing activities in the community [65]. Through collaboration community partners leveraged and optimized resources, shared responsibilities and supported each other’s efforts [65]. At that time, the partnership broadened to other community partners (such as teachers teaching the new curriculum in 1997) [46] thereby extending awareness and commitment to the cause of KSDPP (talking circle, groups 1): “At that time, teachers began to be more comfortable with the new curriculum, and were very committed to the cause”.

An analysis of programming approaches implemented in 1996–1997 reveals that half of the activities were conducted by KSDPP independently whereas half resulted from collaborative partnerships with community organizations [65]. Interestingly, this analysis “found that more than two thirds of collaborations occurred in response to invitations received by KSDPP from other community entities” [65]. In these collaborations, community members and organisations “brought their knowledge of the community, and contributed ideas on how best to carry out the activities in which they were involved” [41]. According to talking circle participants (group 1), trust and respect characterized the relationship with the education system at that time.

### KSDPP’s moment: from 2001 to 2006

Based on its experience in the second stage, KSDPP developed into a stronger organization in the third stage, with well-established partnerships in the community, a well-oiled program of activities and significant community and political recognition. During this period, KSDPP became a leader in Canada for addressing diabetes prevention among First Nations communities [50].

In 2001, KSDPP secured major funding for 5 years from the Canadian Institutes of Health Research (CIHR), permitting the hire of an additional 4 people (including a public relations officer) and the development of the KSDPP Center for Research and Training in Diabetes Prevention [43, 66, 67]. This grant, which acknowledged KSDPP’s experience, expertise and leadership in diabetes prevention and community mobilization, allowed the organization to further community mobilization within Kahnawake, while developing a community mobilization training program to disseminate its intervention model to over 30 Indigenous communities across Canada (from 2001 to 2014) [68]. Inside its own community, KSDPP also reached a high level of credibility owing to its participatory approach, as emphasized by some participants: “I think [that] a lot of the development of KSDPP was done alongside community members so it taught us to have credibility in community” (group 1). “The other organizations within the community have come around recognizing the central role that KSDPP can play in [health promotion and diabetes prevention]” (group 2). At that time, “KSDPP’s visibility in and acceptance by the community suggests that it is perceived as an accessible community resource for health promotion” [65].

During this stage KSDPP’s leaders acquired external recognition from public institutions. For example, in 1999, a KSDPP staff member who was also a community researcher was elected to the Board of Directors of the Canadian National Aboriginal Diabetes Association (NADA), serving as vice-chairperson until 2002 and eventually chairperson from 2002 to 2004. In the years 1999–2001, a physician-researcher deeply involved in KSDPP’s formation and work was elected president of the North American Primary Care Research Group (NAPCRG). She was key in the development of a new policy promoting participatory research in this international organization. In 2010, KSDPP received a Partnership Award from the Canadian Institutes of Health Research for their exemplary work [69]. Even if not specific to the third stage, this award recognized the strength of KSDPP’s work in these times, as well as its contribution to developing ethical agreements with Indigenous communities.

From 2001 to 2006, with funding from the CIHR and the National Aboriginal Diabetes Initiative (Health Canada), KSDPP became active on many levels and continued to extend its reach and vision [41, 70]. As indicated in a scientific article describing KSDPP over this period, “this programme has grown, it has sustained itself and enriched itself in interaction with the community (…)” [41]. KSDPP’s staff disseminated information about the program locally, nationally and internationally by participating in national forums addressing diabetes and health issues for Indigenous people [41]. Inside the community, a KSDPP public relationship office was created to actively disseminate KSDPP’s news through radio shows, newsletters and other means of communication [70] (talking circle, group 1). In 2000, the local Onkwata’karitáhtshera Health and Social Service Research Council was created by the community health board to act as the community ethics board for all health and social research conducted in Kahnawake. This entity acknowledged KSDPP’s CAB as a valid and autonomous ethics authority to evaluate proposals for diabetes prevention research, and added KSDPP’s Code of Research Ethics to its original research agreement terms (talking circles, groups 1 and 2).

At that time, most activities of KSDPP were already collaborative in nature [34], capitalizing on a core of partner organizations that have “taken KSDPP to work together more or less systematically” (talking circle – group 2). They also developed new partnerships with organizations in the private sector of the community, including a local computer software company [66]. Collaborating with new partners allowed “the creation and production of new activities and activity tools (e.g., diabetes awareness booth, cooking demonstrations with students)” [34]. Respect among partners has allowed the program to consistently evolve: “Because each partner’s voice was heard and respected, constructive negotiation occurred allowing transformations in the programme in a way that did not threaten its identity” [41].

KSDPP’s momentum was characterized by the full achievement of its collective action strategy, building on a core program of activities that achieved maturity with the addition of other activity components. A paper describing KSDPP at this period emphasizes that the project “evolved by increasing both the reach and intensity of healthy living interventions” [43]. In addition to the core activities, KSDPP’s program expanded to include preschool children and also engaged adolescents in youth empowerment projects through the community high school [66]. By 2003, there were more than 100 different interventions per year, many in partnership with other community organizations [66]. A descriptive case study of KSDPP at this period highlights that: “There is continuous momentum in active participation of community members involved in diverse activities ranging from research to supporting interventions” [66].

### KSDPP’s maintenance, integration and consolidation: from 2007 to present

The current stage of KSDPP can be characterized by the emergence of a new form of leadership, resource constraints, lower levels of community mobilization and sensitiveness to KSDPP’s message, as paradoxically KSDPP’s vision and goals have become more integrated inside the community and within the agendas and priorities of partner organisations.

Major decreases in funding since 2006 have resulted in the majority of the staff, including the public relations position, retiring from the project. This made it difficult for KSDPP to keep the momentum going in mobilizing the community, as explained by a participant: “(…) To me, [KSDPP brought] very positive changes, but then I guess because of decreased funding and decreased staff, the momentum didn’t keep going” (group 3). According to talking circle participants (group 1), the administrative environment in the community became less supportive of KSDPP activity. Decreases in resources, coupled with a lack of innovation, rendered KSDPP less visible. This phenomenon was highlighted by some community participants (group 4): “When it was very popular, like in the first years… the people knew about it, they were active in schools… Some people didn’t like some of the ideas they were bringing, but it was more known and now it’s very quiet, we don’t hear about it anymore”. An hypothesis evoked is that KSDPP’s action became so integrated into the community that it appeared less noticeable to community members. One participant (group 1) mentioned that “[KSDPP] has become part of the social fabric in the community”, which is, paradoxically, a form of success.

The current stage is characterized by the rise of a new generation of leaders in different parts of the partnership, including the KSDPP research team and KSDPP intervention staff. From a research team perspective, since 2006 the research team has been involved in smaller research projects (many led by postgraduate students, under the supervision of the KSDPP research team) and has included new determinants of diabetes prevention (i.e. food security, adequate sleep) (talking circle, group 2). From a staff perspective, this era is also seen as a turbulent one, with high levels of staff turnover and hiring based on programmatic activity and the availability of funding. New staff members have brought a fresh perspective on the KSDPP collective action strategy and vision, providing renewed energy, all the while ensuring continuity in KSDPP’s overall work (talking circle, group 1). As explained by one participant (group 3): “There have been many different people, different staff over the years, but I see now there are a few new young [people] who work for KSDPP and I see the exact same strength. It’s the way that they’re part of the community and the way that they go and mobilize all their contacts within the community”.

During this stage, the vision promoted by KSDPP (a healthy community, free of diabetes) and the norm underlying this vision (diabetes is a preventable disease) appeared as successfully disseminated in the community. Some participants described this shift in beliefs and norms: “There was a whole change (...), this idea of diabetes being preventable has now become the normal way of thinking…”(group 1). “I remember (...) people coming in and teaching you different things about eating healthier and being healthier and being active, it was sort of like new to us. And now it’s like normal for all the kids to have a nutrition policy in the schools” (group 4). Talking circle participants involved directly in KSDPP (group 1 and 2) were unambiguous about the role the project played in promoting this vision: “KSDPP certainly played the role of that catalyst [for diabetes prevention] in the community” (group 1). “KSDPP was the catalyst to the whole movement. They were the ones that caused this whole spark and this whole awareness and this [desire] to do something about it and the energy that just infiltrated the whole community” (group 2). However, the vision is still not shared by everyone in the community, with some interpreting KSDPP’s message and efforts to implement it as a form of policing: “(…) [some community organizations] have sodas and junk food and things like that in their vending machines. And again, it’s that response ‘It’s our choice to do that” (talking circle, group 1). “I think that there’s part of the population that think that health promotion and diabetes prevention is important but there’s a part of the population that don’t wanna hear about it” (talking circle, group 4).

Regarding the issue of collaboration, KSDPP has allowed many partners to build capacity, and these partners are now taking over some of the responsibilities initially held by KSDPP. For instance, a Masters student research project led to the development and implementation of a physical activity policy in the elementary schools (2011–2013) and a PhD student project conducted in collaboration with a multi-sectorial committee contributed to the development of an active school transportation project (2013–2015). These projects involved representatives of partner organizations, who are now assuming the leadership of these initiatives [71, 72]. A staff member mentioned: “It’s intentionally with everything KSDPP does… we’re working this way, we’re putting ourselves in with everyone else, intentionally trying to mobilize people to take ownership of these issues for themselves” (group 1).

KSDPP’s continuous action has resulted in the integration of its collective action agenda, i.e. fostering healthy eating and physical activity, in some partnering organisations. For instance, the physical activity policy (2011–2013) was developed in close collaboration with the community elementary schools [73]. Participants emphasized the pervasiveness of KSDPP’s agenda on partner organisations: “People have talked about the importance of the wellness policies in the schools and I have a very strong feeling that those would never ever have happened in the early years of KSDPP” (group 2). “KSDPP as a separate entity is able to challenge either the utility of that direction or to explore other areas that perhaps the organisations aren’t focusing on at the moment” (group 1). However, participants (group 1) recognize that there is still resistance from some sectors of the community and some participants (group 2) highlighted the need to build stronger collaborations with some health organizations in the community to get funding instead of competing with each other.

### New proposed benchmarks

Findings from the study point to potentially new benchmarks in the examination and assessment of the development of KSDPP (bolded in Table 3). For instance, in the third stage, a recurrent theme in the “vision and frames” component was broader dissemination of the KSDPP vision and approach across levels of implementation (i.e. local, national and international). This phenomenon has been emphasized both in KSDPP publications over this period, and by KSDPP stakeholders in the talking circles. We therefore propose that broadening dissemination of a project’s vision might be a significant benchmark at this stage. Using the same rationale, additional benchmarks are proposed for stage 3 (Alliances, partnerships, networks; Advocacy agenda and action strategy) and stage 4 (Base building and mobilization; Alliances, partnerships, networks).

### KSDPP’s areas of potential improvement

By comparing the actions and processes of KSDPP to the chosen theoretical framework, this analysis has exposed potential areas of improvement for the initiative.

First, and as emphasized by participants, is the question of continuing leadership: “Looking ahead, [one thing to do] is nurturing the torch bearers for health promotion, diabetes prevention. I don’t know if we have enough of those still generated from KSDPP (…) We served our term and beyond (…) and there needs to be more.” (group 2). Even if some evidence shows a renewing of the research and intervention leadership in KSDPP, there is still some room to plan and foresee the future of the partnership leadership, which is essential in avoiding stagnation or dissipation in a movement. Such an exercise could involve “creating time for intellectual and spiritual reflection by leaders as well as a commitment to training a new generation of leadership” [74].

Second is the need to continuously review and redefine the partnership’s vision and strategies. For instance, one talking circle participant (group 1) suggested broadening the vision and collective action strategy to focus more generally on wellness: “I think one area that we have talked about is the area of wellness in general (…). I think KSDPP started where it was safe, around physical activity and healthy eating (...) we’ve already started to work with stress, mental health and wellness. So is this an area that KSDPP will develop more fully in the future?” Along similar lines, some participants (groups 3 and 4) suggested finding more efficient strategies to ingrain healthy behaviours in children, such as more systematic and direct engagement with parents: “I think sometimes where we miss the mark is that it was aimed primarily at the schools, but it’s the parents who are the role models, it’s the parents who are making the purchases of the food in the home and maybe sometimes there should be more emphasis put on the parents than on the children” (group 3). As suggested by some participants (group 2), renewing KSDPP strategies may also require scaling up or developing further alliances with the political and economic sectors of the community so as to tackle political and systemic determinants of diabetes prevention and health promotion that can’t be addressed by KSDPP alone:

“Something that we talked about (...) is working with the economic sector of the community on health promotion. (...) Because if we look at the people that are selling food, are providing food services, we know that they are supplying demand; the community is demanding salt, fat, sugar, carbs, etcetera. We want them to shift to something else but we always backed off from them.”

The end of this study coincided with KSDPP’S strategic planning exercise (“strategic conversations” with key community actors and members). The first author was invited to participate in the design of these conversations and integrated the results of this study, including potential area of improvement and action paths, in this reflection.

## Discussion

This framework analysis, based on a social movement-building framework [31], portrays the development of KSDPP in a four-stage process of emergence, coalescence, momentum and maintenance/integration; each stage assessed by the achievement of intermediate outcomes, and influenced at different levels and by different kinds of resources, and mobilization, partnership and collective action activities. Based on the framework benchmarks, we conclude that KSDPP has reached the last stage of movement-building, which is the maintenance and integration stage into the Kahnawake community.

Based on this analysis, we can see that KSDPP’s overall reach has expanded from its original vision which was focused on diabetes prevention. Framing KSDPP as a social movement, this study points to other significant processes and outcomes, such as creating awareness; shifting norms and beliefs about diabetes in the community; fostering community mobilization, collaboration and leadership around this issue; building community capacity, skills and expertise in diabetes prevention; creating culture of collaboration and resource sharing among community organizations and permeating the diabetes prevention agenda into other organizations. Previous studies that have looked at KSDPP’s outcomes have tended to provide a mixed picture of the project’s impact on health and the behaviors of residents. One could say that the design of these studies may have failed to capture events and trends in the broader context that influence people’s behaviors and health, such as the introduction of satellite television in the community in 2008, the increasing availability of fast-food restaurants over the last 20 years, as well as strong positive secular trends in the prevalence of obesity [47]. We believe that studies with an exclusive focus on health outcomes pose paradoxes to the very nature of CBPR, which is based on the ecological premise that “an individual’s behavior is shaped by a dynamic interaction with the social environment” [6]. In addition, community-level changes and processes in their own constitute valuable outcomes, and they sometimes have a “more profound impact on well-being than did the intended outcomes of planned interventions” [5]. Our study highlights important community-level processes and outcomes in Kahnawake, which can be considered as transitional steps towards health improvement.

A movement-building framework such as that by Masters and Osborn [31] is an applicable and innovative tool with which to understand and assess CBPR projects. Although the movement-building framework has been applied retrospectively in the current study, it can be used prospectively to encourage ongoing reflection and assessment in the context of CBPR [31]. Using the framework retrospectively can help coalitions situate and assess themselves with respect to the collective action they led and the progress made over the years. Using the framework prospectively can assist coalitions plan ahead by providing general guidance about aspects of the action that are important at a specific moment. While the phases of the framework are modeled on social movement development stages, they nonetheless provide useful markers to assess the development and progress of CBPR projects and other collective action strategies over time, Furthermore, the core concepts of movement-building (i.e. base building and mobilization; leadership; vision and frames; alliances, partnerships, networks; advocacy agenda and action strategy) resonate with the CBPR approach and allow an identification and examination of core CBPR processes and action. Moreover, the benchmarks associated with each phase help identify key accomplishments at each stage as well as areas where additional efforts need to be focused. For instance, it suggests that in the second stage (coalescence) of development, CBPR teams should not expect to pervade the agendas of collaborating organizations, but should rather focus on refining collective action goals; in addition, CBPR leaders should not expect to be recognized from the base, but rather should work at building and expanding core collaboration.

However, while the framework offers a number of distinct intermediate goals on which to focus, it does not provide strategies with which to achieve these goals, which might be a limitation to translating findings into implementation. For example, in the third stage (movement’s moment) of implementation the movement/CBPR project is supposed to see “public support of the meta-narratives increase”, but the framework doesn’t specify how to achieve this benchmark; it only offers examples of trackable progress.

We believe that social movement frameworks, such as the one used in this study, apply particularly well to long-standing, sustainable community-based projects. However, it is important to acknowledge that these frameworks may not be useful or relevant to all CBPR projects. In the case of KSDPP, the specificities of Kahnawake and the Mohawk culture favored the emergence of this form of large, sustainable community-based projects – one that is similar to social movements.

## Conclusion

The current study assessed the processes and intermediate outcomes of the Kahnawake Schools Diabetes Prevention Project using a social movement building framework. This framework analysis describes the development of KSDPP’s in a four-stage process, each stage defined and described by the achievement of important intermediate outcomes and the identification of potential areas of improvement. The framework’s central concepts provide useful markers to situate long-standing and sustainable CBPR projects within its own life course, and inform the development of recommendations to provide guidance for future action. This study proposes some innovative insights regarding the evaluation of CBPR projects and the assessment of their progress by building on their similarities with other forms of collective action.

## Additional file

Additional file 1: List of scientific and organisational documents included in the document review (n = 51). (DOCX 25 kb)

## Abbreviations

CAB: Community Advisory Board
CBPR: Community-based participatory research
CIHR: Canadian Institutes of Health Research
KSDPP: Kahnawake Schools Diabetes Prevention Project
NADA: National Aboriginal Diabetes Association
NAPCRG: North American Primary Care Research Group
