# Supplementary material for: Understanding community-based participatory research through a social movement framework: a case study of the Kahnawake Schools Diabetes Prevention Project
Source: BMC Public Health. 2018 Apr 12;18:487. doi: 10.1186/s12889-018-5412-y (PMC5897940; doi:10.1186/s12889-018-5412-y)
Supplement: Supplementary file 1 — List of scientific and organisational documents included in the document review (n = 51). (DOCX 25 kb) [file 12889_2018_5412_MOESM1_ESM.docx]

**Additional file 1**

| ***Scientific articles*** | |
| --- | --- |
| **No** | **Reference** |
| 1 | Adams A, Receveur O, Mundt M, Paradis G, Macaulay A. Healthy Lifestyle Indicators in Children (Grades 4 to 6) from the Kahnawake Schools Diabetes Prevention Project. Canadian Journal of Diabetes 2005;29(4):402-409. 2005 |
| 2 | Bisset S, Cargo M, Delormier T, Macaulay AC, Potvin L. Legitimizing diabetes as a community health issue: a case analysis of an Aboriginal community in Canada. Health Promot Int 2004;19(3):317-26. |
| 3 | Cargo M, Levesque L, Macaulay AC, McComber A, Desrosiers S, Delormier T, et al. Community governance of the Kahnawake Schools Diabetes Prevention Project, Kahnawake Territory, Mohawk Nation, Canada. Health Promot Int 2003;18(3):177-87. |
| 4 | Cargo M, Salsberg J, Delormier T, Desrosiers S, Macaulay AC. Understanding the social context of school health promotion program implementation. Health Education 2006;106(2):85-97. |
| 5 | Cargo M, Delormier T, Levesque L, Horn-Miller K, McComber A, Macaulay AC. Can the democratic ideal of participatory research be achieved? An inside look at an academic-indigenous community partnership. Health Educ Res 2008;23(5):904-14.08 |
| 6 | Cargo, M, Delormier, T, Levesque, L, McComber, A, Macaulay, AC. Community capacity as an "inside job": Evolution of perceived ownership of a university-Aboriginal community partnership. American Journal of Health Promotion, 26(2), 96-100. 2011. |
| 7 | Delormier T, Cargo M, Kirby R, McComber A. Activity Implementation as a Reflection of Living in Balance: The Kahnawake Schools Diabetes Prevention Project. Pimatisiwin: A Journal of Aboriginal and Indigenous Community Health 2003;1(1):45-163. |
| 8 | Hogan L., Garcia-Bengoechea E., Salsberg J., Jacobs J., King M., Macaulay AC. Using a participatory approach to the development of a school-based physical activity policy in an Indigenous community. Journal of School Health, 2014. |
| 9 | Horn OK, Paradis G, Potvin L, Macaulay AC, Desrosiers S. Correlates and predictors of adiposity among Mohawk children. Prev Med 2001;33(4):274-81. |
| 10 | Horn OK, Jacobs-Whyte H, Ing A, Bruegl A, Paradis G, Macaulay AC. Incidence and prevalence of type 2 diabetes in the First Nation community of Kahnawa:ke, Quebec, Canada, 1986-2003. Can J Public Health 2007;98(6):438-43. 2007 |
| 11 | Hovey R., Delormier T., McComber A. Social-relational understandings of health and well-being from an Indigenous Perspective. International Journal of Indigenous Health, 2014. |
| 12 | Jimenez MM. Evaluation of dietary change among Kahnawake Schools Diabetes Prevention Project participating children (grades 4-6) [Thesis M Sc]. McGill University, 2000. |
| 13 | Jimenez MM, Receveur O, Trifonopoulos M, Kuhnlein H, Paradis G, Macaulay AC. Comparison of the dietary intakes of two different groups of children (grades 4 to 6) before and after the Kahnawake Schools Diabetes Prevention Project. J Am Diet Assoc 2003;103(9):1191-4. |
| 14 | Levesque L, Cargo M, Salsberg J. Development of the Physical Activity Interactive Recall (PAIR) for Aboriginal children. Int J Behav Nutr Phys Act 2004;1(1):8. |
| 15 | Levesque L, Guilbault G, Delormier T, Potvin L. Unpacking the black box: a deconstruction of the programming approach and physical activity interventions implemented in the Kahnawake Schools Diabetes Prevention Project. Health Promotion Practice 2005;6(1):64-71. |
| 16 | Macaulay AC, Montour LT, Adelson N. Prevalence of diabetic and atherosclerotic complications among Mohawk Indians of Kahnawake, PQ. CMAJ 1988;139(3):221-4. |
| 17 | Macaulay AC. University-Community Collaboration in Primary Care Research. In: MJ Bass, EV Dunn, PG Norton, M Stewart, F Tudiver, editors. Conducting Research in the Practice Setting. Thousand Oaks: Sage Publications, 1991. |
| 18 | Macaulay AC, Paradis G, Potvin L, Cross EJ, Saad-Haddad C, McComber A, et al. The Kahnawake Schools Diabetes Prevention Project: intervention, evaluation, and baseline results of a diabetes primary prevention program with a native community in Canada. Prev Med 1997;26(6):779-90.ulay 1997 |
| 19 | Macaulay A, Paradis G, Potvin L, Cross E, Saad-Haddad C, McComber A, et al. Primary Prevention of Diabetes Type II in First Nations: Experiences of the Kahnawake Schools Diabetes Prevention Project. Can J Diabetes Care 1998;22:44 - 49. |
| 20 | Macaulay AC, Cross EJ, Delormier T, Potvin L, Paradis G, McComber A. Developing a Code of Research Ethics for research with a Native community in Canada: a report from the Kahnawake Schools Diabetes Prevention Project. Int J Circumpolar Health 1998;57 Suppl 1:38-40. |
| 21 | Macaulay AC, Delormier T, McComber AM, Cross EJ, Potvin LP, Paradis G, et al. Participatory research with native community of Kahnawake creates innovative Code of Research Ethics. Can J Public Health 1998;89(2):105-8. |
| 22 | Macaulay AC, Commanda LE, Freeman WL, Gibson N, McCabe ML, Robbins CM, et al. Participatory research maximises community and lay involvement. North American Primary Care Research Group. BMJ 1999;319(7212):774-8. |
| 23 | Macaulay AC, Harris SB, Lévesque L, Cargo M, Ford E, Salsberg JS, et al. Primary Prevention of Type 2 Diabetes: Experiences of Two Aboriginal Communities in Canada. Canadian Journal of Diabetes 2003;27(4):464-475. |
| 24 | Macaulay AC, Cargo M, Bisset S, Delormier T, Levesque L, Potvin L, et al. Community Empowerment for the Primary Prevention of Type 2 Diabetes: Kanien'keha:ka (Mohawk) ways for the Kahnawake Schools Diabetes Prevention Project. In: Ferreira ML, Lang GC, editors. Indigenous Peoples and Diabetes: Community Empowerment and Wellness. Durham, NC: Carolina Academic Press, 2006. |
| 25 | Macaulay AC, Ing A, Salsberg J, McGregor A, Rice J, Montour L, et al. Community-based participatory research: sharing results with the community. An example of knowledge translation from the Kahnawake Schools Diabetes Prevention Project. Progress in Community Health Partnerships: Research, Education, and Action 2007;1(2):143-152. |
| 26 | Macridis S, Garcia Bengoechea E, McComber AM, Jacobs J, Macaulay AC, Members of the Kahnawake Schools Diabetes Prevention Project-School Travel Planning Committee. Active transportation to support diabetes prevention: Expanding school health promotion programming in an Indigenous community. Evaluation and Program Planning 56 (2016), 99-108. 2016.is 2016 |
| 27 | McComber AM, Macaulay AC, Kirby R, Desrosiers S, Cross EJ, Saad-Haddad C. The Kahnawake Schools Diabetes Prevention Project: community participation in a diabetes primary prevention research project. Int J Circumpolar Health 1998;57 Suppl 1:370-4. |
| 28 | Mercille G, Receveur O, Macaulay AC. Are snacking patterns associated with risk of overweight among Kahnawake schoolchildren? Public Health Nutr 2009:1-9. |
| 29 | Montour LT, Macaulay AC. High prevalence rates of diabetes mellitus and hypertension on a North American Indian reservation. Can Med Assoc J 1985;132(10):1110, 1112. |
| 30 | Montour LT, Macaulay AC, Adelson N. Diabetes mellitus in Mohawks of Kahnawake, PQ: a clinical and epidemiologic description. CMAJ 1989;141(6):549-52. |
| 31 | Paradis G, Levesque L, Macaulay AC, Cargo M, McComber A, Kirby R, et al. Impact of a diabetes prevention program on body size, physical activity, and diet among Kanien'keha:ka (Mohawk) children 6 to 11 years old: 8-year results from the Kahnawake Schools Diabetes Prevention Project. Pediatrics 2005;115(2):333-9. |
| 32 | Potvin L, Desrosiers S, Trifonopoulos M, Leduc N, Rivard M, Macaulay AC, et al. Anthropometric characteristics of Mohawk children aged 6 to 11 years: a population perspective. J Am Diet Assoc 1999;99(8):955-61.tvin 1999 |
| 33 | Potvin L, Cargo M, McComber AM, Delormier T, Macaulay AC. Implementing participatory intervention and research in communities: lessons from the Kahnawake Schools Diabetes Prevention Project in Canada. Soc Sci Med 2003;56(6):1295-305. |
| 34 | Receveur O, Morou K, Gray-Donald K, Macaulay AC. Consumption of key food items is associated with excess weight among elementary-school-aged children in a Canadian first nations community. J Am Diet Assoc 2008;108(2):362-6. |
| 35 | Salmon L. Contribution of foods to nutrient intakes of grades 4--6 students participating in Kahnawake Schools Diabetes Prevention Project, 1994, 1998 and 2002 [Masters Thesis]. McGill University, 2004. |
| 36 | Salsberg J, Louttit S, McComber A, Fiddler R, Naqshbandi M, Receveur O, et al. Knowledge, Capacity and Readiness: Translating Successful Experiences in CBPR for Health Promotion. Pimatisiwin: A Journal of Indigenous and Aboriginal Community Health 2008;6(1). |
| 37 | Salsberg J, Macridis S, Garcia Bengoechea E, Macaulay AC, Moore S, Members of the Kahnawake Schools Diabetes Prevention Project - School Travel Planning Committee. Engaging Community Stakeholders for School-Based Physical Activity Intervention. Retos - Nuevas Tendencias en Educacion Fisica, Deporte y Recreacion, Vol. 28. 2015. |
| 38 | Trifonopoulos M. Anthropometry and diet of Mohawk schoolchildren in Kahnawake [Thesis M Sc]. McGill, 1995. |
| 39 | Trifonopoulos M, Kuhnlein HV, Receveur O. Analysis of 24-hour recalls of 164 fourth- to sixth-grade Mohawk children in Kahnawake. Journal of the American Dietetic Association 1998;98(7):814-6. |
| ***Organisational documents*** | |
| 1 | Kahnawake Schools Diabetes Prevention Project. KSDPP Schools & Community Intervention Activities for 2016-2017, Organisational document, KSDPP: Kahnawake. |
| 2 | Kahnawake Schools Diabetes Prevention Project. KSDPP Monthly Activities for 2010-2011, Organisational document, KSDPP: Kahnawake. |
| 3 | Kahnawake Schools Diabetes Prevention Project. KSDPP Annual Summary for 2010-2011, Organisational report, KSDPP: Kahnawake. |
| 4 | Kahnawake Schools Diabetes Prevention Project. KSDPP Workplan, Breakdown month by month for 2014-2015, Organisational document, KSDPP: Kahnawake. |
| 5 | Kahnawake Schools Diabetes Prevention Project. KSDPP Workplan, Breakdown month by month for 2013-2014, Organisational document, KSDPP: Kahnawake. |
| 6 | Kahnawake Schools Diabetes Prevention Project. KSDPP Workplan, Breakdown month by month for 2012-2013, Organisational document, KSDPP: Kahnawake. |
| 7 | Kahnawake Schools Diabetes Prevention Project. KSDPP Schools & Community Intervention Activities for 2015-2016, Organisational document, KSDPP: Kahnawake. |
| 8 | Kahnawake Schools Diabetes Prevention Project. ADI Community Project Summary for 2011-2012, Organisational report, KSDPP: Kahanwake. |
| 9 | Kahnawake Schools Diabetes Prevention Project. KSDPP school summaries of activities for 2011-2012, Organisational report, KSDPP: Kahnawake. |
| 10 | Kahnawake Schools Diabetes Prevention Project. Community Monthly Activities for 2009-2010, organisational document, KSDPP: Kahnawake. |
| 11 | Kahnawake Schools Diabetes Prevention Project. School summaries of activities for 2009-2010, organizational document, KSDPP: Kahnawake. |
| 12 | Kahnawake Schools Diabetes Prevention Project. Summaries of activities for 2008-2009, Organisational report, KSDPP: Kahnawake. |
